# Exercise-induced osteoimmune crosstalk: a virtuous cycle between bone remodeling and immune regulation

**DOI:** 10.3389/fendo.2026.1904464

**Published:** 2026-07-29

**Authors:** Linlin Wang, Weiying Guo

**Affiliations:** Department of Endocrine and Metabolic Diseases, The First Hospital of Jilin University, Changchun, Jilin, China

**Keywords:** bone metabolism, exercise, immune system, osteoimmunology, virtuous cycle

## Abstract

As an interdisciplinary field investigating the skeletal and immune systems, bone immunology has revealed that these two systems share the bone marrow microenvironment, interacting with and regulating each other. Exercise, as a non-pharmacological intervention, has been demonstrated to provide a favorable environment for bone homeostasis by modulating the dynamic balance between osteogenesis and bone resorption. Furthermore, exercise enhances the body’s anti-inflammatory capacity by regulating various immune components, including immune cells and cytokines, thereby optimizing overall immune function. This article systematically reviews how exercise may initiate a feed-forward osteoimmune circuit in which mechanical loading activates osteocytes and osteogenic progenitors, reshapes the bone marrow niche, promotes anti-inflammatory immune polarization, and subsequently favors bone formation over bone resorption Ultimately, this creates a virtuous cycle of bone immunology, offering novel therapeutic strategies for the prevention and treatment of bone metabolic diseases such as osteoporosis.

## 1 Introduction

The term “osteoimmunology“ was coined by Arron and Choi (1), to describe an interdisciplinary field investigating the bidirectional regulation between the skeletal and immune systems. These two systems share a common origin—the bone marrow microenvironment (2). For instance, immune cells modulate osteoclast and osteoblast activity via secreted cytokines (3), while cell surface receptors (e.g., Toll-like receptors) recognize pathogen-associated molecular patterns (PAMPs) or damage-associated molecular patterns (DAMPs) to regulate bone metabolism (4). Conversely, osteoblasts and osteoclasts can influence immune cell production (5). This intricate interplay not only maintains bone homeostasis but is also implicated in various pathological conditions. As an emerging research frontier, bone immunology holds promise for developing novel therapeutic strategies for metabolic bone diseases.

Against this backdrop, exercise, as the most accessible physiological stimulus, represents a non-pharmacological intervention. Many bone cells are mechanosensitive (6), and the conversion of mechanical forces generated by human movement into bone loads serves as the key stimulus for maintaining bone homeostasis (7). Concurrently, exercise serves as an effective immunomodulator; appropriate physical activity can enhance immune competence and attenuate chronic inflammation, which is closely associated with exercise intensity, duration, age, and other factors (8). Based on this, we hypothesize that appropriate exercise, bone, and immunity form a virtuous cycle. More specifically, mechanical loading acts as the primary stimulus to regulate immune cells and associated factors, thereby inducing immune remodeling; this remodeled immunity subsequently further optimizes the bone microenvironment, which in turn feeds back to modulate the immune system. Through this dual action, bone homeostasis is maintained.

Current research predominantly examines the interactions between osteocytes and immune cells, as well as the isolated effects of mechanical loading on bone metabolism and immune function. However, this body of work largely conceptualizes bone-immune crosstalk as a static, bidirectional molecular dialogue—overlooking critical features such as feedback regulation, temporal dynamics, and systemic integration. In contrast to prior reviews, which treat bone and immune processes as parallel yet disconnected phenomena, this review introduces the novel integrative framework of the “mechano-immuno-bone axis. “We posit that physical movement does not exert merely two independent beneficial effects—”enhancing skeletal integrity” and “modulating immune competence”—but rather orchestrates bone remodeling and immune regulation through shared mechanotransduction pathways, thereby constituting a unified, adaptive physiological response. Within this framework, bone is reconceptualized not as a passive structural scaffold but as an active “mechanical–immune sensor,” offering a biologically grounded perspective on the evolutionary and functional interdependence of the musculoskeletal and immune systems. Importantly, this contribution represents theoretical integration—not *de novo* discovery—yet we argue that synthesizing fragmented empirical observations into a coherent, dynamic, and predictive model constitutes a substantive conceptual advance. Specifically, the framework enables testable predictions: for instance, it allows differentiation of how distinct mechanical stimuli—such as high-frequency/low-amplitude versus low-frequency/high-amplitude loading—differentially engage the mechano-immuno-bone axis. Ultimately, this paradigm shift holds translational promise, informing mechanism-informed therapeutic strategies for metabolic bone disorders. This review systematically integrates current evidence to propose a feed-forward osteoimmune circuit initiated by mechanical loading, offering a mechanistic framework for exercise-based interventions in metabolic bone diseases.

## 2 Mechanical loading and bone remodeling

### 2.1 Exercise and bone formation

Exercise-induced mechanical stress triggers bone-building responses, improving bone structure and mineral density. The extracellular matrix (9), integrins (10), and primary cilia (11) are key sensors in this process, converting mechanical signals into cellular actions. At the molecular level, evidence suggests exercise prevents bone marrow stem cells from turning into fat cells, instead encouraging them to become bone-forming cells (12). Through these sensors, mechanical stress from exercise boosts the differentiation of these stem cells into bone cells by regulating factors like Signal Transducer and Activator of Transcription 3 (STAT3) (7), Long non-coding RNA H19 (lncRNA H19) (13), and immediate early response 3 (IER3) (14). Moreover, it can increase E2a expression by reducing p21 levels, further promoting bone cell growth and division (15).

In terms of signaling pathways, Mechanical stress enhances cell proliferation and promotes the osteogenic differentiation rate of ligamentum flavum ossification cells by regulating the bone morphogenetic protein–Smad1 (BMP-Smad1) and Src-p38 mitogen-activated protein kinase (Src-p38MAPK) signaling pathways (16). Concurrently, it activates the release of osteogenesis-related markers such as osteocalcin and alkaline phosphatase, thereby facilitating osteogenesis. Additionally, Exercise also increases miR-154-5p levels, which targets Wnt11 to enhance the osteogenic differentiation of fat-derived stem cells through the Wnt/PCP pathway (17). Notably, the Wnt/β-catenin pathway is vital for bone formation. Clinical research shows that 8 weeks of resistance training notably boosts Wnt pathway proteins, including β-catenin, in teenagers (18), indicating its high sensitivity to exercise-induced mechanical loads.

From a hormonal perspective, animal studies reveal that acute resistance exercise fosters bone growth through various endocrine pathways. It boosts insulin-like growth factor-1 (IGF-1), triggering autophagy by blocking the PI3K/Akt/mTOR pathway (19). Exercise also adjusts aromatase activity, enhancing local estrogen production (20). Higher estrogen levels prevent bone cell death, extend their lifespan, and upregulate bone-forming markers like osteocalcin and collagen (21, 22). Additionally, estrogen improves integrin-based mechanotransduction and activates key signaling pathways, collectively enhancing bone formation and metabolism (23).

### 2.2 Exercise and bone resorption

#### 2.2.1 Direct inhibition of osteoclasts

Short-term mechanical stress directly hinders osteoclast development by reducing the mRNA levels of specific genes involved in their formation at an early stage (24), The RANKL/RANK/OPG system is key in regulating osteoclast differentiation and activity (25). *In vivo* research shows that acute, moderate exercise lowers RANKL, a marker of bone breakdown, in mice (26). *In vitro*, oscillating fluid flow, mimicking natural mechanical stress, decreases the RANKL/OPG ratio by reducing RANKL and boosting OPG, thereby curbing osteoclast production (27). Because RANKL can be produced not only by osteoblast-lineage cells but also by activated immune cells, exercise-induced immunomodulation may indirectly affect osteoclastogenesis through immune-cell-derived RANKL.

#### 2.2.2 Indirect regulation of osteoclasts

Osteocytes, when mechanically stimulated, secrete interleukin-6 (IL-6), steering RAW264.7 cells away from becoming osteoclasts and lowering osteoclast marker expression in precursor cells, thus curbing RANKL-triggered bone breakdown (28). They also hinder monocyte movement via primary cilia and PTH1R, reducing osteoclast recruitment (29). Moreover, mechanical force lets osteocytes block human monocyte differentiation into osteoclasts through CAV-1, while boosting stem cell migration (30); Exercise also increases the secretion of the myokine L-β-aminoisobutyric acid (L-BAIBA), which suppresses osteoclastogenesis by inhibiting the PI3K/AKT/NF-κB pathway (31).

### 2.3 Effects of exercise on bone homeostasis

**Figure 1:**
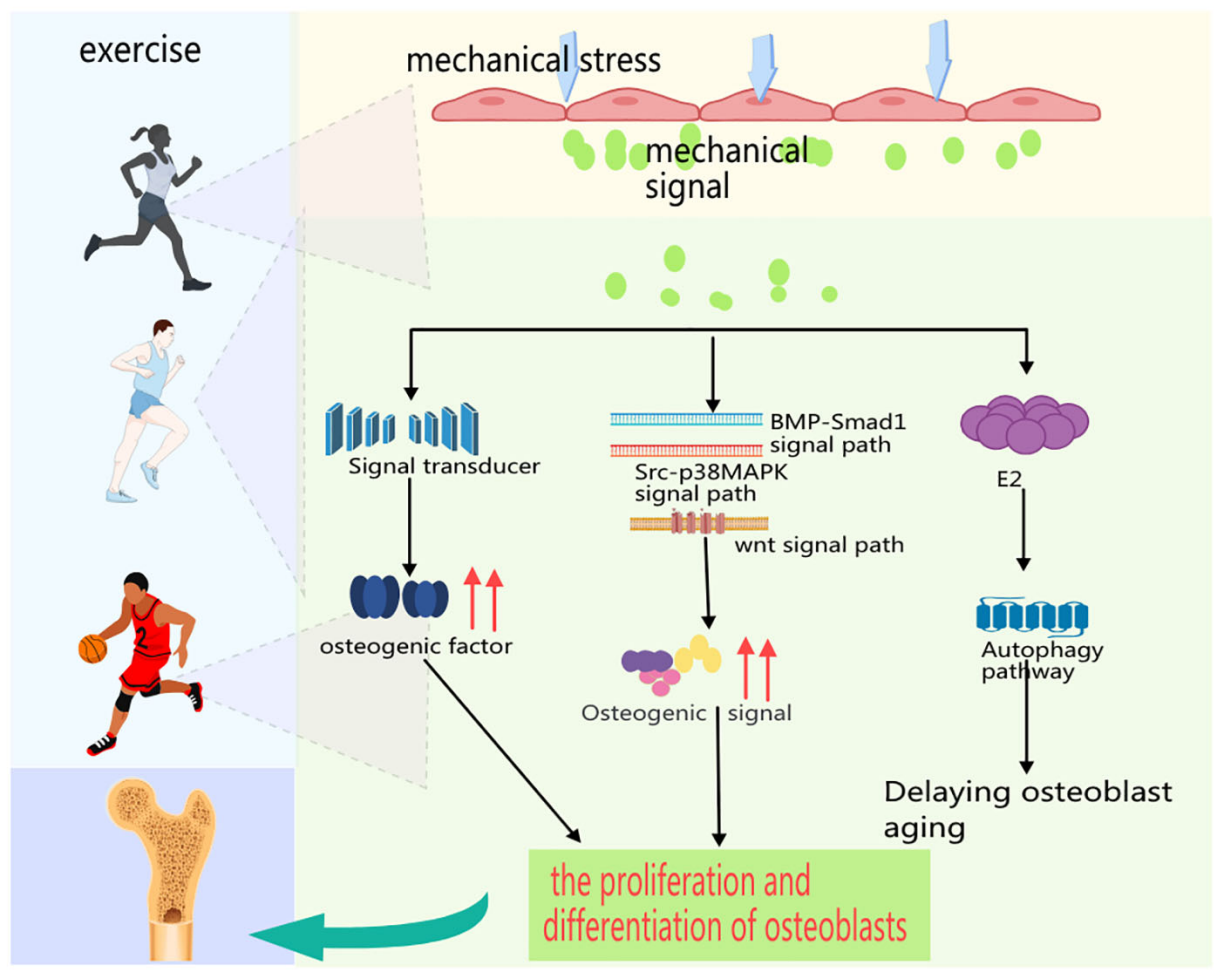
Exercise-induced mechanical loading promotes osteogenesis through mechanotransduction pathways.

**Figure 2:**
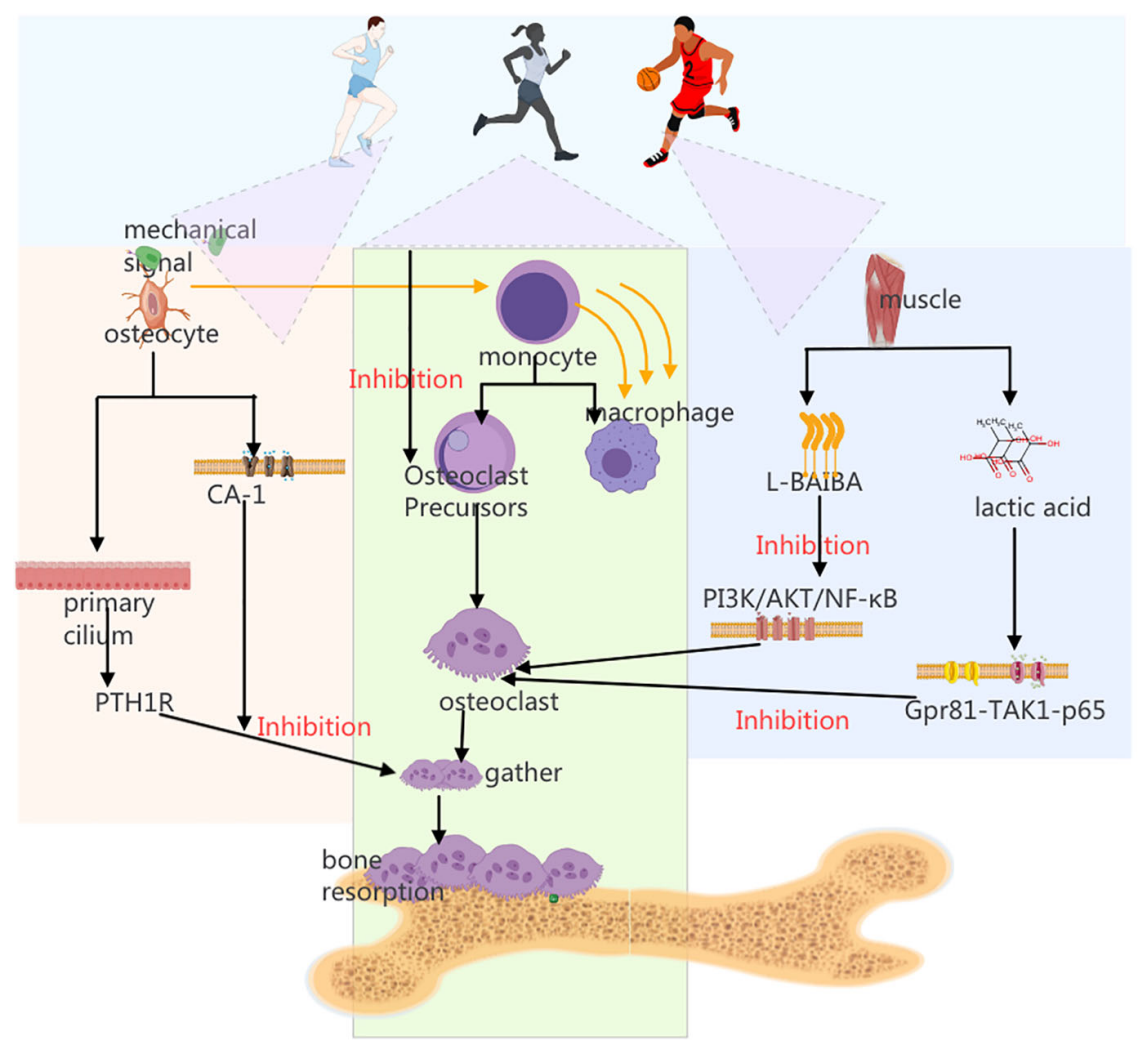
Exercise inhibits bone resorption by generating mechanical and biochemical signals that suppress osteoclastogenesis.

In conclusion, exercise can promote bone formation (**Figure 1**) and inhibit bone resorption (**Figure 2**) through various aspects such as mechanical stimulation, signal pathways, and hormone regulation, achieving the goal of positively regulating bone metabolism and maintaining bone homeostasis. This brings new treatment strategies for bone diseases related to bone homeostasis imbalance. However, mechanistic studies on exercise and bone metabolism have been largely confined to animal experiments, with relatively scarce clinical evidence. And during human movement, cells are not only affected by fluid shear stress, but also endure compressive strain, hydrostatic pressure, and matrix deformation, among other complex forces. The temporal dynamic characteristics of these processes are further regulated by systemic factors such as changes in blood flow, hormonal fluctuations, and neural input, which are completely absent in cell culture. Therefore, attributing the effect of exercise in regulating bone metabolism solely to the action of a certain pathway is a significant inference that requires careful interpretation. On the other hand, the regulatory effect of exercise on bone homeostasis is not invariably favorable. Clinical evidence indicates that long-term endurance athletes may exhibit lower bone mineral density compared to sedentary controls, suggesting that excessive training loads could tip the balance toward bone resorption predominating over bone formation (32). Conversely, meta-analytic evidence has indicated that resistance exercise exerts a significant osteogenic effect, as reflected by increased bone mineral density (33). This apparent paradox likely stems from divergent loading characteristics inherent to distinct exercise modalities. Endurance training is characterized by low-magnitude, high-frequency repetitive loading that may favor osteoclast-mediated bone resorption, whereas resistance training involves high-magnitude, low-frequency loading that may preferentially stimulate osteoblast-mediated bone formation (34). Moreover, energy availability may be a key confounder—endurance athletes often experience low energy availability, whereas resistance trainers typically maintain energy sufficiency. Yet few studies have rigorously controlled for both mechanical and energetic variables, limiting the comparability of conclusions. Hence, exercise exerts variable effects on bone homeostasis, and the optimal “exercise dose” for each individual is that which effectively promotes bone formation without precipitating excessive stress.

## 3 Exercise-induced immune remodeling

### 3.1 The impact of acute exercise on the immune system

#### 3.1.1 For immune cells directly

Acute exercise induces a rapid and transient lymphocytosis, characterized by increased circulating lymphocyte counts (35). Concurrently, exercise promotes the mobilization and redistribution of natural killer (NK) cells into peripheral blood (36). Acute exercise preferentially mobilizes cytotoxic T cells, thereby enhancing immune surveillance against viral pathogens (37).

From the perspective of metabolic mechanisms, at the onset of acute exercise, the sympathetic-adrenal medullary (SAM) axis is rapidly activated (38), triggering catecholamine secretion. These catecholamines activate α1-adrenergic receptors (α1-ARs) (39), thereby suppressing tumor necrosis factor (TNF) (40) production in inflammation-stimulated cells and exerting anti-inflammatory effects. Beyond quantitative expansion, acute exercise enhances the capacity of immune cells to secrete anti-inflammatory factors (41). Furthermore, exercise preferentially mobilizes cytotoxic T cells and nonclassical monocytes into the bloodstream, redeploying these effector cells to protect the host from potential damage or infection associated with acute stress responses (42).

From the perspective of myogenic cytokine anti-inflammation, acute exercise induces transient intensification of skeletal muscle contraction, triggering robust secretion of myokines, including IL-6 (43), interleukin-8 (IL-8) and interleukin-15 (IL-15) (44). IL-6 can also directly act on the adrenal medulla, promoting cortisol release—a potent anti-inflammatory glucocorticoid (45). On the other hand, the increased IL-15 can promote the self-renewal of CD8T cells when progenitor cells are exhausted during continuous antigen promotion (46), and also promote the survival of CD8T cells and the cytotoxic effector phenotype (47). Beyond these myokines, intense exercise upregulates peroxisome proliferator-activated receptor-γ coactivator-1α (PGC-1α) expression in skeletal muscle (48). PGC-1α subsequently drives the transcription of genes encoding anti-inflammatory mediators, including IL-10 and transforming growth factor-β (TGF-β), thereby bolstering immune homeostasis (49).

### 3.2 The impact of long-term exercise on the immune system

#### 3.2.1 Long-term exercise enhances immune function

Long-term exercise can enhance the mucosal immune function of the elderly (50) and attenuates the production of inflammatory biomarkers (51), thereby alleviating the systemic inflammatory response. Exercise also modulates the hematopoietic niche, reprograms hematopoietic stem and progenitor cells, and curtails inflammatory leukocyte production (52), ultimately enhancing immune competence.

#### 3.2.2 Long-term exercise optimizes the ability of immune cells

Long-term training expands the NK cell pool while maintaining these cells in a quiescent state with reduced resting cytotoxicity (53). Long-term exercise also attenuates adipose tissue inflammation by suppressing classically activated (M1) macrophage accumulation and promoting alternative (M2) macrophage polarization. This phenotypic switch is mediated by enhanced M2-polarizing transcription factor activity and epigenetic silencing of M1-associated gene loci (54, 55). Furthermore, exercise augments neutrophil responsiveness, enhancing chemotactic and phagocytic functions (56).

#### 3.2.3 Long-term exercise delays immune aging

Clinical evidence demonstrates that long-term cyclists exhibit elevated circulating thymopoietic factors, which attenuate thymic involution and reduce T cell senescence (57)At the cellular level, telomere length—protective caps at chromosome termini that dictate cellular replicative lifespan—serves as a critical biomarker of immunosenescence (58). Elite endurance athletes display preserved telomere length, indicating that exercise maintains chromosomal integrity and delays immune cell aging (59). Regular exercise also increases the concentration of betaine. Betaine reduces chronic inflammation and delays immune aging by inhibiting TBK1 (60). Consistent with these findings, animal studies reveal that exercise diminishes reactive oxygen species (ROS) accumulation in aged mice, restores CD8+ T cell proportions, and attenuates systemic immunosenescencee (61). Collectively, long-term exercise delays immune cell aging through multiple convergent pathways.

### 3.3 Exercise and immunity

**Figure 3:**
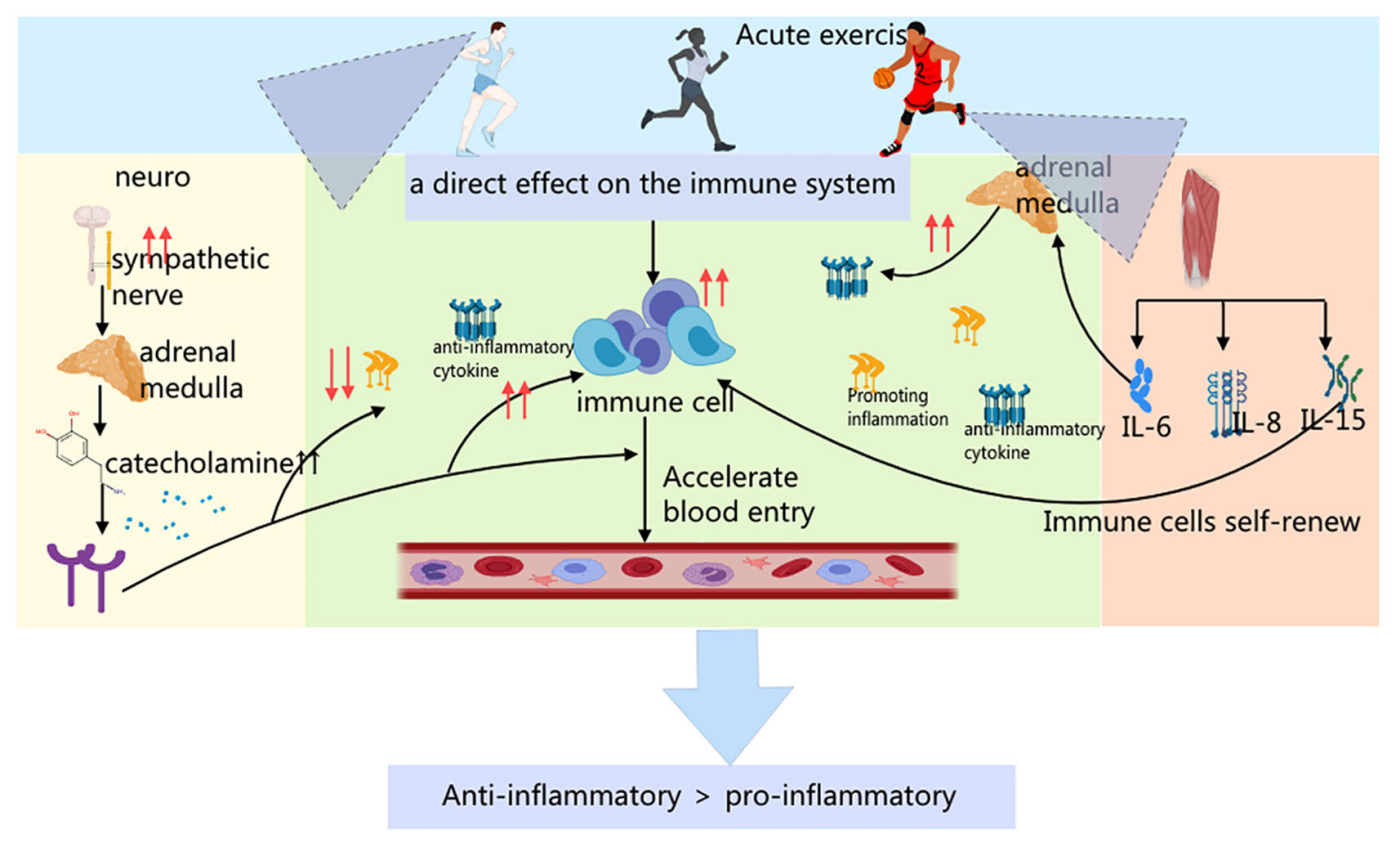
The impact of acute exercise on the immune system.

**Figure 4:**
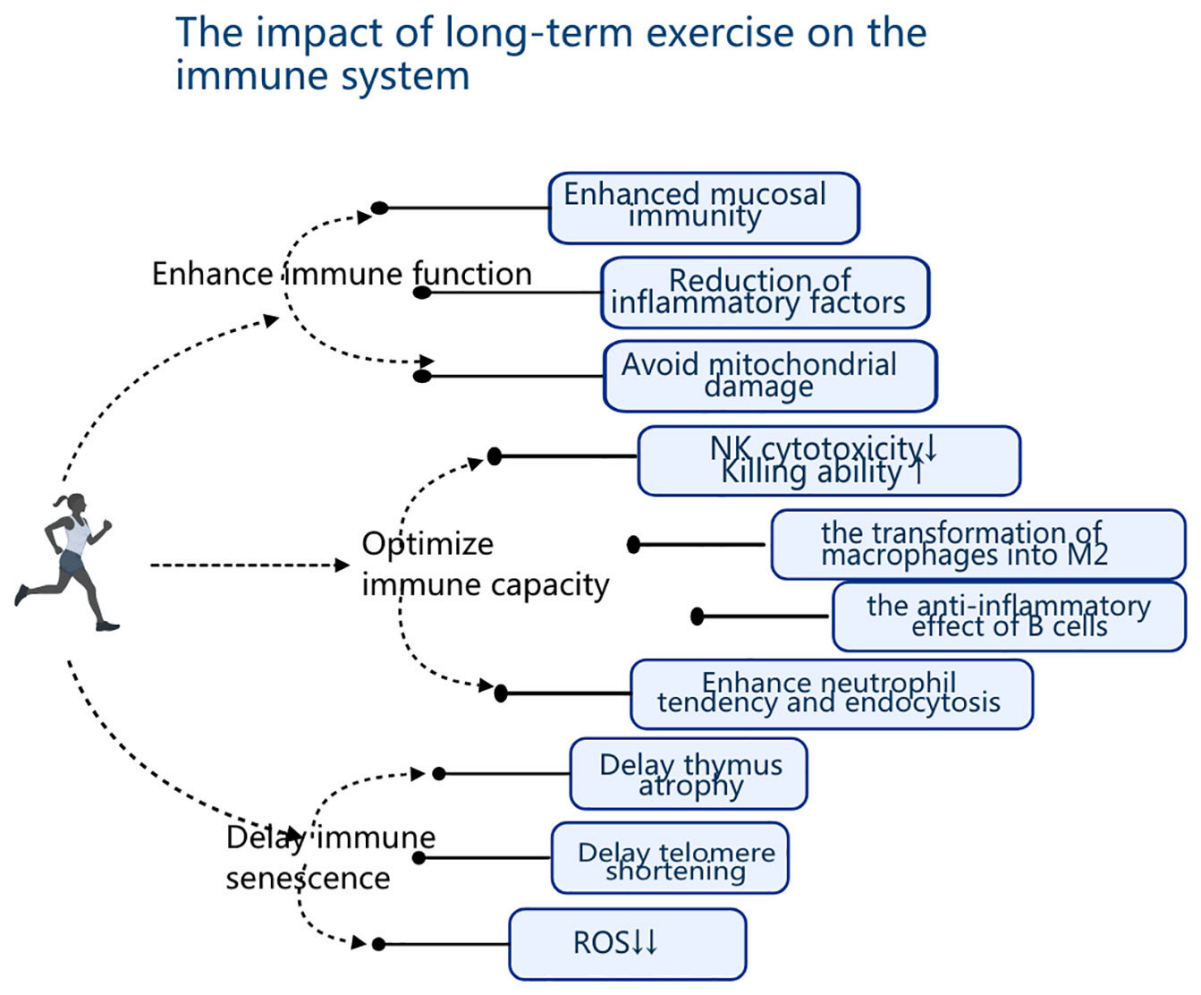
The impact of long-term exercise on the immune system.

On the one hand, acute exercise kicks the immune system into gear—immune cells surge, the adrenal medullary system fires up fast, and overall, the body’s anti-inflammatory power ramps up. We also see muscles and bones talking to the immune system, shifting the secretion of immune cytokines (**Figure 3**): On the other hand, long-term exercise can enhance and optimize the ability of immune cells, delay immune aging, and strengthen immune function (**Figure 4**). In conclusion, regular moderate exercise generally promotes an anti-inflammatory immune milieu, whereas prolonged strenuous exercise or insufficient recovery may induce transient immune perturbation and inflammatory stress. However, the exercise modalities and intensities employed in the aforementioned studies warrant further investigation. Moreover, evidence suggests that long-term high-intensity exercise may induce immunosuppression (62). Acute high-intensity exercise can also trigger a burst of oxidative stress, leading to elevated inflammatory cytokines, exacerbated infection risk, and compromised immune function (63). Notably, current immunological studies have been largely circumscribed to peripheral blood monitoring, notwithstanding that immune reactions are fundamentally tissue-resident processes and exhibit a time-dependent dynamic evolution. However, the temporal resolution of exercise-mediated immune regulation has yet to be systematically characterized. Thus, the immune consequences of exercise defy oversimplification, and a considerable number of unresolved issues remain.

## 4 The virtuous cycle effect of exercise on osteoimmunology

### 4.1 Osteoimmune alterations under special conditions

#### 4.1.1 Age-related decline of the osteoimmune axis

During the aging process, the bone microenvironment and the immune system exhibit synergistic degeneration, characterized by progressive fatty replacement of the bone marrow cavity that encroaches upon hematopoietic space, diminished osteoblastic differentiation capacity, and reduced mechanosensory function of the osteocyte network (64). Concurrently, thymic involution leads to reduced naive T cell output, accumulation of memory T cells, and elevated baseline levels of pro-inflammatory cytokines, collectively establishing a state of “inflammaging” (65). This dual decline impairs the transduction efficiency of exercise-induced mechanical signals into immunomodulatory signals, necessitating a higher threshold or prolonged mechanical stimulation in the elderly population to elicit comparable osteoimmune responses. However, the causal direction between inflammaging and bone loss remains unclear—specifically, whether systemic inflammation drives bone resorption or whether bone-derived inflammatory signals accelerate immunosenescence. Existing studies are predominantly cross-sectional in design, lacking the longitudinal data required for causal inference.

#### 4.1.2 Osteoimmune axis remodeling under injury and disease conditions

Osteoimmune axis remodeling under injury and disease conditions can be summarized as a dual pathological pattern consisting of a local vicious cycle of inflammation and bone resorption, alongside systemic immunosuppression coupled with impaired tissue repair (66). Following trauma, pro-inflammatory cytokines (TNF-α, IL–1β, and IL–6) released from damaged tissues activate osteoclasts, whereas effector memory CD8^+^T cells suppress osteogenic differentiation of mesenchymal progenitor cells via IFN-γ and TNF, thereby exacerbating healing impairment (67). In rheumatoid arthritis (RA), as a representative disease, the Th17 cell-IL-17-RANKL axis is overactivated, leading to excessive osteoclastogenesis (68). Meanwhile, TNF induces the expression of DKK1 and sclerostin, which inhibit Wnt-mediated osteogenic signaling, resulting in a “high resorption-low formation” paradox (69). The bone–immune interaction is bidirectional; pathological bone conditions such as osteopetrosis can directly lead to immunodeficiency by disrupting the hematopoietic bone marrow microenvironment. Therefore, osteoimmune axis remodeling under injury and disease fundamentally represents a positive feedback loop in which bone resorption-formation imbalance and immune activation-suppression imbalance mutually reinforce each other.

### 4.2 Bone marrow niche as the hub connecting exercise, bone and immunity

#### 4.2.1 From the perspective of basic substances: enhancing vascular structure and function

A meta-analysis shows that exercise increases the number of hematopoietic stem cells in the body (70), changing how the bone marrow works and speeding up blood cell regeneration (71). Clinical research goes a step further, showing that regular workouts boost the release of both bone marrow hematopoietic cells and endothelial progenitor cells linked to VEGF and interleukin-6 (72). After just 30 minutes of running, levels of VEGFR2+ endothelial progenitor cells jump noticeably (73). All of this points to a simple truth: exercise strengthens hematopoiesis and helps blood flow better (74).

On the other hand, it was experimentally verified that treadmill exercise induces a marked upregulation of VEGF in rats, thereby facilitating bone vascularization. Angiogenesis bone marrow is a complex process; notably, mechanical loading has been shown to enhance angiogenesis, with particularly significant increases in the expression of sphingosine 1-phosphate receptor 1 (S1PR1) in H-type vessels (75). Consequently, this provides more abundant nutritional and oxygen supply while offering an expanded niche for hematopoietic stem cells. In conclusion, exercise creates a more oxygen- and nutrient-rich microenvironment in the bone marrow by enhancing hematopoiesis and improving circulation. Most evidence linking exercise or mechanical loading to type H vessel expansion is derived from animal or controlled-loading models; whether similar vascular remodeling occurs in humans after different exercise modalities remains insufficiently established.

#### 4.2.2 From a spatial perspective: expand the favorable space of the bone marrow

The accumulation of bone marrow adipose tissue is closely associated with the suppression of hematopoietic function (76). From the perspective of origin, cell experiments have shown that exercise can prevent bone marrow adipose tissue formation in mice fed a high-fat diet (77). Regarding molecular mechanisms, mechanical stimulation can inhibit adipogenic differentiation of bone marrow mesenchymal stem cells (BMSCs) by downregulating peroxisome proliferator-activated receptor gamma (PPARγ), thereby promoting osteogenic differentiation and reducing marrow cavity adiposity (78). This not only provides sufficient space for the growth of various cell types but also creates a more favorable microenvironment for hematopoiesis, innervation, and vascularization (79). Furthermore, Exercise may reduce marrow adiposity and alter lipid availability within the marrow niche; however, whether marrow adipocyte-derived lipids directly fuel hematopoiesis, osteoblast activity, or lymphopoiesis requires further experimental validation (80).

#### 4.2.3 From the cellular perspective: coordinate cell mobilization

From an anti-inflammatory perspective, exercise can reduce circulating inflammatory cytokines, alleviate bone marrow inflammation, facilitate the quiescence and normal function of bone marrow cells, and optimize the immune system. Exposure of cells to mechanical stretching of different magnitudes revealed that mechanical stretch confers protection on BMSCs via activation of AMPK phosphorylation, thereby attenuating mitochondrial ROS accumulation (81). From the perspective of aging, long-term exercise enhances metabolism and maintains bone and muscle health through epigenetic modifications, including DNA hypomethylation, histone modifications, and non-coding RNA network remodeling (82), maintaining the bone marrow in an undifferentiated, primitive state and delaying stem cell exhaustion.

In summary, exercise can enhance bone marrow perfusion, increase oxygenation, provide abundant nutrients, promote hematopoiesis, and suppress inflammatory factors, thereby reducing bone marrow cell senescence and decreasing senescence-associated metabolites. This creates a bone marrow microenvironment that tends toward anti-inflammatory, antioxidant, nutrient-rich, and rejuvenated states, consequently improving the capacity of bone to regulate systemic organ function.

### 4.3 Exercise regulates bone metabolism through the immune system

#### 4.3.1 From a cellular perspective

Macrophage bidirectional regulation: The mouse treadmill exercise model has been proven that mechanosensitive ion channel 1(Piezo1) can induce macrophage polarization toward anti-inflammatory phenotypes by mediating mechanical loading, i.e., enhancing M2 macrophage polarization. These M2 macrophages promote the proliferation, migration, and osteogenic differentiation of bone marrow mesenchymal stem cells (BMSCs) through TGF-β1 secretion (83). Additionally, enhanced M2 polarization increases exosome secretion, which inhibits TNF-α production and thereby suppresses RANKL-induced osteoclast differentiation, ultimately regulating bone metabolism (84).

Precise regulation of T cells: Animal experiments have proved that regular aerobic exercise can increase the number of Treg cells in the body (85), Treg cells can inhibit osteoclast differentiation and maintain bone mass by producing TGF-βand IL-4 (86), Exercise can also reduce the differentiation of Th17 cells by inhibiting ROR-γt (87), which leads to a decrease in the secretion of IL-17 and the expression of RANKL thereby inhibiting osteoclast differentiation (88). In summary, under inflammatory or metabolic stress conditions, regular moderate exercise appears to shift the Th17/Treg balance toward an anti-inflammatory profile, which may reduce IL-17/RANKL-driven osteoclastogenesis.

#### 4.3.2 Exercise maintains bone homeostasis through a balance between anti-inflammation and pro-inflammation

Clinical studies have shown that lifelong athletes maintain elevated levels of anti-inflammatory molecules, such as TGF-β and IL-10 (89). Animal experiments have further demonstrated that moderate exercise increases systemic levels of various anti-inflammatory factors, including IL-10, IL-4, moderate levels of IFN-γ, and TGF-β (90). This establishes a fundamental anti-inflammatory microenvironment, thereby modulating downstream signaling cascades. For instance, elevated IL-10 inhibits NF-κB pathway activation by phosphorylating and activating the JAK1/STAT3 axis (91), consequently attenuating osteoclastogenic signals (92). Additionally, increased TGF-β signaling occurs primarily through SMAD family proteins. TGF-β activates both canonical and non-canonical SMAD pathways, which converge at the transcription factor RUNX2 to promote osteoblast differentiation and maturation (93).

Exercise regulates bone metabolism by regulating bone-related factors secreted by various immune cells. Exercise can also regulate the interaction between osteogenesis and osteoclasts in an anti-inflammatory microenvironment by increasing the secretion of anti-inflammatory factors, maintaining bone homeostasis.

### 4.4 Exercise regulates the immune system by its impact on bone homeostasis

#### 4.4.1 Bone cells sense mechanical signals to recruit immune cells

The results in the mouse model showed that mature osteocytes sense mechanical stimulation through Piezo1, induce the activation of akt, and thereby inhibit sclerotin (94), The inhibition of sclerotin reduces its effect on the differentiation of Th17 cells and enhances the differentiation of Treg cells, tilting the immune system towards anti-inflammation and thereby reducing Inflammatory bone destruction (95).

#### 4.4.2 Exercise mediates the regulation of the immune system by bone-derived hormones

Bone tissue functions as an endocrine organ capable of modulating systemic metabolism and immunity. Exercise enhances osteogenic activity and promotes the secretion of bone-derived hormones. A clinical study demonstrated that postmenopausal women with osteopenia exhibited significantly elevated circulating osteocalcin levels following regular exercise (96). This elevated osteocalcin has been shown to suppress the secretion of pro-inflammatory cytokines, including tumor necrosis factor-α (TNF-α) and interleukin-6 (IL-6), while concurrently stimulating the production of the anti-inflammatory cytokine interleukin-10 (IL-10) *in vitro* (97). Additionally, regular exercise induces an increase in circulating osteopontin (OPN), which subsequently upregulates transforming growth factor-β1 (TGF-β1) secretion, thereby modulating immune function (98). Exercise also enhances the expression of osteoprotegerin (OPG), leading to increased production of interleukin-2 (IL-2) and interleukin-4 (IL-4) (99). Furthermore, exercise-stimulated bone formation promotes osteoblast secretion of chemokines, such as C-X-C motif chemokine ligand 12 (CXCL12), which optimizes the bone marrow hematopoietic stem cell niche, facilitates the proliferation and differentiation of immune cell precursors (e.g., lymphoid progenitor cells), and enhances immune cell generation efficiency (100, 101). Collectively, these findings indicate that exercise modulates the bone marrow microenvironment, regulates immune homeostasis, and attenuates inflammatory cell production (52).

In summary, bone perceives the mechanical stress generated by exercise, activates osteoblasts, and upregulates osteocalcin levels, thereby influencing the secretion of related inflammatory cytokines, tilting the entire immune system toward an anti-inflammatory state, promoting the generation and expression of immune cells, and enhancing the body’s immune capacity.

**Figure 5:**
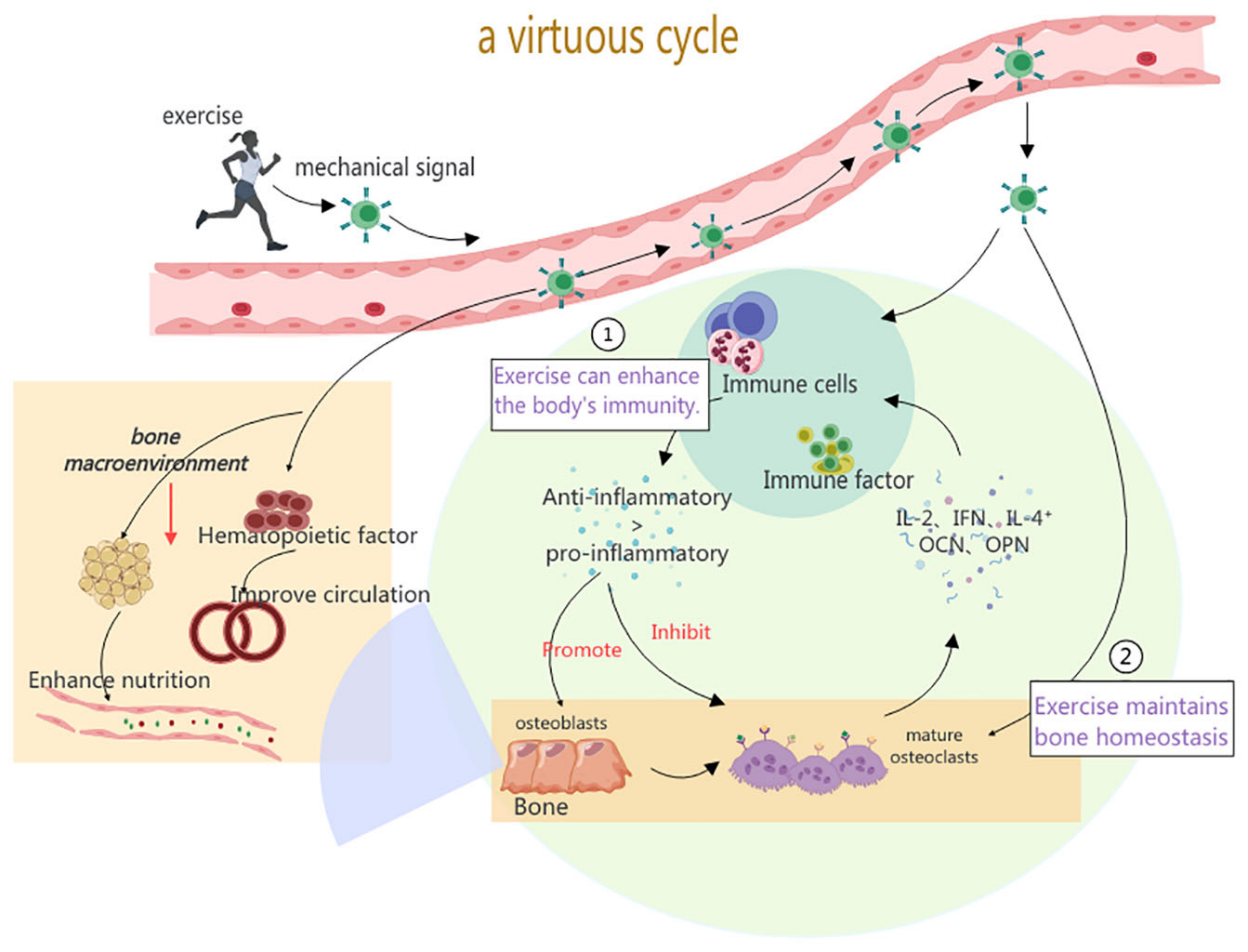
Exercise promotes a virtuous cycle of osteoimmunology.

At this point, a complete virtuous cycle is established (**Figure 5**). Exercise can promote the generation of immune cells and optimize immune function, enhancing the body’s immune capacity, The optimized immune function (Treg cells ↑, M2 macrophages ↑, anti-inflammatory factors ↑, pro-inflammatory factors ↓) can promote bone formation while inhibiting bone resorption, maintain bone homeostasis, and create a healthy bone microenvironment, A good bone microenvironment, in turn, strengthens vascular structure and improves circulation. The chemokines released by bone cells further enhance the immune capacity and improve the bone’s regulatory ability over all organs of the body, enabling the body to benefit from exercise. Form a virtuous cycle of exercise-immune-bone homeostasis.

## 5 Clinical implications and exercise prescription

### 5.1 Effects of exercise modality and intensity on osteoimmunology

#### 5.1.1 Exercise dose-response relationship

Although exercise independently elicits nonlinear dose-response effects on bone metabolism and immune function, the integrated dose-response relationship at the osteoimmune axis level has received scant attention. From a theoretical perspective, the relationship between exercise dose and osteoimmune benefits is likely to follow an inverted U-shaped trajectory (102). The state of physical inactivity is characterized by decreased bone formation, relatively increased bone resorption, bone marrow adiposity (103), elevated baseline levels of pro-inflammatory cytokines, and accelerated immunosenescence (104). In the moderate-intensity exercise zone (hypothetically the optimal zone), mechanical loading activates osteocyte signaling pathways, enhances osteoblast differentiation, elevates anti-inflammatory cytokine levels, and potentiates regulatory T cell (Treg) function. In this zone, it is hypothesized that TGF-β and osteocalcin released during bone formation support Treg differentiation, thereby establishing a positive feedback loop. However, this mechanistic framework has thus far been derived solely from animal model-based hypotheses and has not been corroborated by clinical evidence. The excessive-intensity exercise domain is typified by a negative bone remodeling balance—resorption surpassing formation—accompanied by acute elevations in serum CTX, cortisol-driven apoptosis of osteoblasts, and consequent heightened susceptibility to stress fractures (105). Concurrently, acute immunosuppression ensues, manifesting as a transient “open window” period lasting 3 to 72 hours post-exercise, during which peripheral blood lymphocyte counts decline and susceptibility to opportunistic infections increases (106).

#### 5.1.2 Sex-specific differences

Sex-dependent disparities constitute a non-negligible factor in exercise-mediated osteoimmune regulation. Notably, basal Toll-like receptor (TLR) expression in skeletal muscle has been reported to be approximately 24% higher in females than in males. Furthermore, the immune response to non-habitual exercise exhibits substantial sex-related divergence, with specific signal transduction components—such as TLR1, TLR8, TRIF, and IKKβ—being altered solely in males in response to resistance training (106). These observations indicate that the innate immune system in females possesses a higher baseline sensitivity to exercise stimuli, albeit with sex-selective engagement of downstream effector pathways. Moreover, the osteoclast-inhibitory action of estrogen predisposes postmenopausal women to a more fragile osteoimmune equilibrium, and the immunomodulatory outcomes of exercise are plausibly modulated by the prevailing estrogen milieu (107). In males, testosterone serves as the predominant androgen, with dihydrotestosterone playing a secondary role. Testosterone directly promotes osteoblast proliferation via the androgen receptor and supports bone mineralization (108). Developing differentiated exercise strategies for different genders has a significant biological basis. Currently, the research on gender differences in exercise intervention is seriously imbalanced. Most RCTs include post-menopausal women, while data on men, young women, and transgender individuals are scarce.

### 5.2 Exercise interventions for different populations

#### 5.2.1 Adolescents: immune shaping during the bone mass accrual window

Adolescence represents a critical window for bone mass accrual (109). and also serves as a vital stage for immune system maturation (110). Animal studies have demonstrated that exercise can stimulate the bone marrow microenvironment by activating the mechanosensitive ion channel Piezo1 on osteocytes, thereby supporting the development of B lymphocytes and T (111). However, the translational evidence for these mechanistic pathways in humans remains notably sparse—indeed, no investigation to date has performed simultaneous assessments of bone remodeling indices and bone marrow immunological endpoints in adolescent cohorts following controlled exercise protocols. We propose that the osteoimmune integration status in adolescents may be effectively monitored through a panel of biomarkers encompassing: (1) bone metabolic indices—serum OC and BALP as indicators of bone formation activity; (2) immune parameters—peripheral blood IL-6 (differentiating acute versus chronic elevations), IL-10, and TNF-α; and (3) developmental measures—height, weight, and Tanner stage. Nevertheless, this conceptual framework currently lacks empirical validation and awaits experimental testing.

#### 5.2.2 Postmenopausal women: addressing estrogen withdrawal-induced osteoimmune dysregulation

Postmenopausal women face a dual osteoimmune crisis precipitated by the precipitous decline in estrogen levels. Estrogen deficiency upregulates RANKL expression, thereby enhancing osteoclast activity (112), ultimately resulting in bone resorption exceeding bone formation and increasing the risk of osteoporosis. This has been validated in both animal and clinical studies. Exercise intervention demonstrates unique osteoimmune regulatory value in this population. An evidence-based study has demonstrated that when postmenopausal women engage in exercise for ≤6 months with a single-session duration of ≤60 minutes, bone resorption markers, specifically CTX, exhibit a significant decrease, while bone formation markers show a marked increase (113). Furthermore, exercise may promote the synthesis of myogenic estrogen by enhancing the expression of estrogen precursors and steroidogenic enzymes in skeletal muscle (20). However, this study was of a cross-sectional design and thus unable to establish a causal relationship; moreover, its primary endpoint was myogenic estrogen, while the effects on bones and immunity were inferred indirectly.

#### 5.2.3 Elderly population: addressing the dual decline of bone and immune function

Aging is frequently accompanied by the synchronous progression of declining bone formation capacity and immunosenescence (114, 115). The central paradox in exercise intervention for elderly individuals lies in the fact that mechanical stimulation of sufficient intensity is a prerequisite for activating the bone-immune cycle, whereas high-impact exercise concurrently increases the risk of falls and fractures. Animal models have shown that whole-body vibration can affect the function of bone cells through mechanical sensitive channels (116). However, the immunomodulatory effects of vibration training in humans remain largely uninvestigated. Future studies are warranted to elucidate: (1) whether vibration parameters (frequency, amplitude, and duration) are sufficient to activate the same signaling pathways in humans; (2) whether age-related differences in sensitivity exist; and (3) whether vibration training exerts any impact on circulating progenitor cell mobilization and T-cell receptor repertoire diversity—for which no data are currently available.

#### 5.2.4 Athletes: balancing high bone turnover and immune homeostasis

Athletes exhibit a state of high bone turnover, and long-term high-intensity training poses distinctive challenges to the bone-immune system. Overtraining syndrome is characterized by persistently elevated inflammatory markers, Th1/Th2 cell ratio imbalance, and a negative balance wherein bone resorption exceeds bone formation (117, 118). However, most of these studies are cross-sectional in design and thus cannot establish causality. Periodized training design—characterized by the fluctuating arrangement of training loads to avoid the sustained accumulation of mechanical stress and metabolic burden—is critical for maintaining osteoimmune homeostasis in athletes. We propose the following indicators as a conceptual monitoring framework for future research: osteoimmune surveillance in athletes should be integrated into routine physical examination protocols, with particular emphasis on serum sclerostin levels as a reflection of recent mechanical loading history, and osteocalcin fragments as dynamic markers of bone turnover.

### 5.3 Knowledge gaps

#### 5.3.1 Current research limitations

Although research on the promotion of the bone-immune positive cycle by exercise has made some initial progress, the existing studies have significant flaws and the clinical application faces multiple difficulties. A substantial body of evidence has been derived from *in vitro* mechanical loading experiments or anesthetized animal models, in which the isolated mechanical stimuli differ fundamentally from the complex physiological context of human exercise. Exercise entails not only mechanical factors but also the integration of multiple systemic processes, including metabolic fluctuations, hormonal rhythms, neural regulation, and psychological stress. Existing studies have thus been limited in their ability to isolate the independent contribution of mechanical loading. Furthermore, the bone remodeling cycle spans approximately 3 to 6 months, whereas immune parameters fluctuate on a timescale of hours to days. This temporal mismatch poses a considerable challenge to the elucidation of synchronous mechanisms. Moreover, the causal relationship between bone and immunity remains difficult to establish in existing studies. Most of the current evidence is derived from cross-sectional designs, which are inherently limited in their ability to control for confounding factors such as diet, sleep, training history, and baseline hormonal status. The available data are largely correlational in nature, and few studies have attempted to manipulate bone status to observe subsequent immune changes, or conversely, to modulate immune function to verify skeletal responses. As a result, the causal direction between bone and immune systems remains an open question, trapped in a circular reasoning loop.

#### 5.3.2 Clinical translation challenges

The clinical translation of exercise-promoted bone-immune virtuous cycles continues to encounter several critical bottlenecks. The precise delineation of optimal exercise dosage represents the foremost challenge. Existing evidence indicates that intensity, frequency, and duration exhibit interactive effects, with dose-response curves varying significantly across different populations, necessitating the establishment of individualized models based on baseline bone-immune phenotypes. The differential activation of bone-immune pathways by distinct exercise modalities remains incompletely elucidated: resistance training predominantly engages the mTOR/IGF-1 axis, aerobic exercise activates the AMPK-SIRT1 pathway, and vibration training targets the Piezo1 mechanosensitive channel, with the temporal synergistic effects of combined applications yet to be revealed. Furthermore, challenges persist in evaluating exercise efficacy as a pharmaceutical intervention. Beyond conventional bone formation markers, bone resorption markers, and inflammatory cytokines, bone marrow microenvironmental markers and mechanical loading indicators collectively constitute a multidimensional assessment system for bone-immune health. The integration of wearable devices with point-of-care testing technologies holds promise for achieving real-time feedback between exercise loading and bone-immune responses, thereby advancing the digital management paradigm of “exercise as medicine”.

## 6 Conclusion

Exercise fosters a virtuous cycle between bone and immune systems, strengthening the intimate connection between the skeletal and immune systems. Within the bone microenvironment, this establishes a ternary theory of mechanical stimulation–bone metabolism–immune regulation, enabling the development of scientifically grounded exercise guidelines tailored to bone-immune mechanisms for diverse populations and bone-immune-related diseases. This approach enhances skeletal health across the entire lifespan, thereby promoting deep interdisciplinary integration among sports medicine, immunology, and bone biology, and catalyzing the emergence of “Exercise Osteoimmunology” as a novel subspecialty. Future endeavors must consolidate multidisciplinary expertise spanning molecular biology, biomechanics, immunology, and clinical medicine to decipher the individualized code of exercise dosage, elucidate the molecular basis of pathway divergence, and establish a quantifiable clinical assessment framework. Ultimately, this will standardize exercise intervention as a therapeutic modality for the prevention and treatment of bone-immune-related diseases, benefiting broad populations from adolescence to senescence, and from health maintenance to disease rehabilitation.
